# A Two-Stage Screw Detection Framework for Automatic Disassembly Using a Reflection Feature Regression Model

**DOI:** 10.3390/mi14050946

**Published:** 2023-04-27

**Authors:** Quan Liu, Wupeng Deng, Duc Truong Pham, Jiwei Hu, Yongjing Wang, Zude Zhou

**Affiliations:** 1 School of Information Engineering, Wuhan University of Technology, Wuhan 430070, China; 2 Department of Mechanical Engineering, University of Birmingham, Birmingham B15 2TT, UK

**Keywords:** robotic disassembly, screw detection, illumination condition, reflection feature, data learning

## Abstract

For remanufacturing to be more economically attractive, there is a need to develop automatic disassembly and automated visual detection methods. Screw removal is a common step in end-of-life product disassembly for remanufacturing. This paper presents a two-stage detection framework for structurally damaged screws and a linear regression model of reflection features that allows the detection framework to be conducted under uneven illumination conditions. The first stage employs reflection features to extract screws together with the reflection feature regression model. The second stage uses texture features to filter out false areas that have reflection features similar to those of screws. A self-optimisation strategy and weighted fusion are employed to connect the two stages. The detection framework was implemented on a robotic platform designed for disassembling electric vehicle batteries. This method allows screw removal to be conducted automatically in complex disassembly tasks, and the utilisation of the reflection feature and data learning provides new ideas for further research.

## 1. Introduction

Remanufacturing is part of a circular economy, returning end-of-life (EOL) products to at least like-new conditions through a group of operations, beginning with disassembly [1,2]. The benefits of remanufacturing for the environment, society, and economy have been widely confirmed through reducing carbon emissions, energy consumption, etc. [3,4,5,6]. Recently, research on remanufacturing electric vehicle (EV) batteries has attracted much attention. As an environmentally friendly option for transportation, the proportion of EVs to all sold cars has increased considerably in the last two years across the world. However, this increase has led to the rapid disposal of EV components, which is also a threat to the environment, society, and economy. Among these components, EV batteries are valuable for recycling because of their expensive and hazardous materials (e.g., lithium and cobalt). In addition, the lifespan of EV batteries is approximately 10 years, which also intensifies the need for remanufacturing them with the growing demand for EVs [7].

Disassembly is the first and inevitable operation in the remanufacturing process due to stricter environmental regulations and growing demand for effective product remanufacturing [8,9,10]. In current applications, manual disassembly is still the main strategy, which consumes a large amount of energy and exposes operators to dangerous materials. The utilisation of robotics has been considered to be an important method in realising automatic disassembly because of its intelligent perception system and effective execution system [11]. As the most widely used perception system, robot vision can automatically investigate the structural information of EOL products and contributes to the decision making in the disassembly process, such as disassembly sequence planning [12,13]. However, there are still some challenges in disassembly tasks for robot vision, mainly caused by the uncertainty and complexity of EOL products [14]. The structural information of EOL products is usually changed and difficult to estimate, which is a barrier to setting detection criteria. In addition, 2-dimensional (2D) cameras are the mainly adopted sensors in robot vision systems, and 2D images are widely used to accurately and efficiently describe the structural information of EOL products. However, the image quality is closely related to the lighting conditions. It is difficult to create a stable lighting condition, which is significant for accomplishing disassembly tasks stably and accurately. The study of robot vision is ponderable for disassembly.

**Figure 1:**
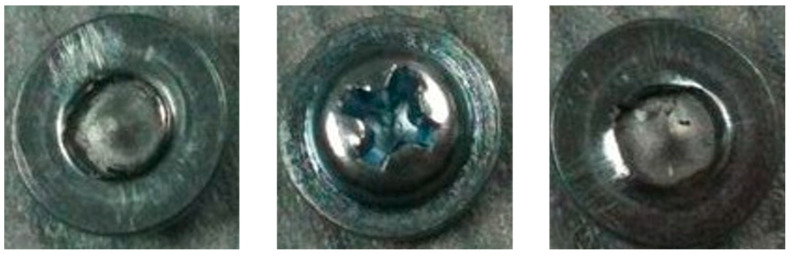
Examples of used and structurally damaged screws.

Screw detection and removal are necessary for almost all EOL products, and the structures of used screws are usually damaged, such as cracks, fractures, and wear and tear, as shown in Figure 1. The uncertain conditions of used screws pose challenges to stable and accurate detection in disassembly tasks. In existing screw detection methods, the detection criteria are mainly designed and concluded based on the texture features of the original screws or a small number of used screws. The performance of these methods in disassembly tasks is limited due to the following challenges:

(1) The texture features obtained from the original screws cannot characterise the used screws accurately due to the unavoidable structural damage during the use of the product.
(2) The robustness of texture features extracted from a small number of available used and damaged screws is limited due to the uncertain conditions of used screws.
(3) The texture features are easily affected by illumination conditions. Current detection methods cannot operate stably under uneven illumination conditions.

To address these issues, this paper presents a novel screw detection method containing a two-stage detection framework and a reflection feature regression model to detect structurally damaged screws in EOL products under uneven illumination conditions. The contributions of this paper are as follows:

(1) It presents a robust feature descriptor for structurally damaged screws by integrating reflection features and texture features. This is beneficial in weakening the influence of structural damage on modelling screws.
(2) It proposes a linear regression model that enables the reflection features to be updated based on the illumination conditions automatically, which contributes to the stable operation of feature modelling under various illumination conditions. In addition, an illumination label is defined to measure illumination conditions automatically and conveniently from the point of view of the image.
(3) It details a two-stage detection framework based on the proposed feature descriptor. With the help of the linear regression model, the two-stage detection framework can extract used screws from EOL products under uneven illumination conditions.

Based on the proposed screw detection method, automatic screw removal was realised for an EOL EV battery using a robotic disassembly platform. The experimental results demonstrate the satisfactory accuracy and stability of the proposed screw detection method.

The remainder of this paper is organised as follows. Section 2 reviews the visual detection work in disassembly cases. Section 3 introduces the proposed screw detection method. Section 4 describes the equipment and experiments, while the results are discussed in Section 5. Section 6 summarises this paper and lists future work.

## 2. Related Work

### 2.1. Screw Detection for Disassembly

In disassembly tasks, screw detection plays a vital role by providing the position for later disassembly. The existing screw detection methods can be classified into experience-based methods and data-driven methods. Experience-based methods utilise prior product information provided by skilled operators to set detection criteria and then extract the screw. Knowledge and model are the most widely used types of experience because they are user-friendly to summarise. Gli et al. [15] set detection criteria based on screw contour information and adopted well-known strategies (e.g., polygonal approaches) to detect screws from EOL Computers. Bdiwi et al. [16] utilised Kinect to collect and characterise screws in the form of greyscale, depth, and HSV values and then proposed a three-stage screw detection framework for removing screws from EOL EV motors. DiFilippo et al. [17] combined Gaussian blur, Prewitt edge detection, region erosion, and Hough and circle detection methods in screw detection, which successfully removed 96.5% of the screws from EOL laptops. The parameters in the above methods were all determined by the structural information of the original screws. Under this situation, the developed criteria cannot represent used screws well due to the uncertainty of EOL products, and the detection criteria are also hard to conclude because of the complexity of EOL products.

The detection criteria of data-driven methods are obtained through data learning represented by deep learning models. In existing studies, researchers are focusing on utilising and optimising state-of-the-art models. DiFilippo et al. [18] designed a cognitive architecture based on Soar’s long-term and semantic memory function, which performed well in determining the label and position of screws in laptops. Using this cognitive architecture, the inference time was decreased by up to 60% and the average inference time was decreased by 10% for most EOL laptops. Foo et al. [19] employed the residual network (ResNet) in unfastening crosshead screws from EOL LCD monitors and achieved optimal precision and recall rates of 91.8% and 83.6%. Mangold et al. [20] used the you only look once (YOLO) model to detect screws in a vision-based robotic disassembly platform. The achieved mean average precision was around 0.98. During the application of these models, some strategies (e.g., transfer learning and dropout) were adopted to reduce the randomness of training. However, these detection models require a large amount of labelled data in the training process, which poses great challenges to disassembly tasks. The robustness of the deep learning models is unsatisfactory due to the uncertainty of the EOL products. There is no guarantee that the feature extractor trained on poorly diversified datasets can accurately characterise the unseen EOL products.

Some researchers have combined experience-based methods and data-driven methods. Li et al. [21] proposed an accurate screw detection method based on the faster region with convolutional neural network features (R-CNN) model and rotation edge similarity, where faster R-CNN is a deep learning model and rotation edge similarity is designed based on the former experience provided by operators. This strategy improves the robustness of the detection method and reduces the number of training data used. It also reached up to a 90.8% success rate in the disassembly process. The proposed screw detection method is based on this strategy.

On the other hand, existing studies designed detection criteria mainly based on texture features, which are easily changed and difficult to estimate in used screws. Furthermore, the lighting condition has not been fully considered, which is also an important problem in utilisation. These two problems are considered in this study, where a robust feature descriptor is designed to characterise the used screws.

### 2.2. Reflection Feature for Object Detection

Reflection features can be used to represent illumination conditions and object characteristics. Currently, reflection removal and detection are the two main methods used in the object detection field. In reflection removal, reflection features are regarded as noises that damage object characteristics. Here, researchers proposed various reflection removal methods mainly based on retinex theory, which assumes that an image can be decomposed into reflection and illumination components [22,23,24]. In reflection detection, reflection features are utilised as intrinsic features. Sudo et al. [25] proposed a glass detection method by focusing on the reflective properties of the glass surface. Wu et al. [26] used reflection features to track and locate moving objects in videos and proved the contribution of reflection features to detecting non-line-of-sight objects. Zhang et al. [27] presented a reflective learning model, and the reflection features were extracted to detect salient objects. Zhang et al. [28] also employed reflection features in designing a loss function to learn the saliency feature in object detection.

In summary, reflection features play a vital role in characterising objects, especially in some cases where texture features cannot perform well. However, previous studies seldom considered the impact of illumination conditions on reflection features, which is important in industrial tasks. This paper presents a reflection feature regression model to automatically determine the reflection feature based on the illumination conditions.

## 3. Method

**Figure 2:**
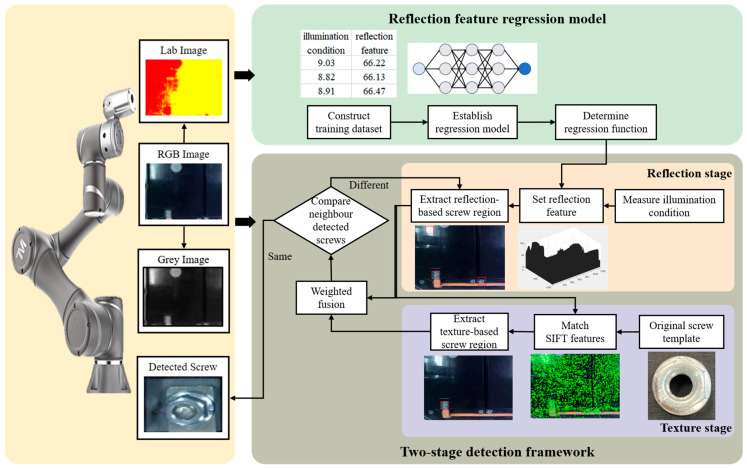
Flowchart of the proposed screw detection method.

This paper proposes an illumination-adaptive detection method for removing screws from EOL products, as shown in Figure 2. In the form of screw region extraction, this method can stably detect structurally damaged screws under nonuniform illumination conditions. The main idea is to characterise and merge the reflection and texture features of the screw regions in the two-stage detection framework. The reflection features are utilised here to model the overall screw regions and then employed to extract screw regions in the reflection stage, which is composed of the measure illumination condition node, the set reflection feature node, and the extract reflection-based screw region node. Compared with texture features, reflection features are less affected by structural damage and can be used to represent structurally damaged screws [29]. In the texture stage, texture features are employed to remove false areas (e.g., exposure areas) extracted before, through the match scale-invariant feature transform (SIFT) features node and the extract texture-based screw region node. The extracted screws of the two stages are then fused in the weighted fusion node, where the screw region with the lowest fused detection confidence is named the detected screw. The detected screw is continuously updated based on a self-optimisation strategy through the compare neighbour detected screws node. Here, the problem of detecting damaged screw structures under a fixed illumination condition is solved. However, in disassembly tasks, controlling lighting conditions is difficult and labour-intensive. To improve the robustness of the proposed detection method under uneven illumination conditions, a reflection feature regression model is developed to draw the relationship between reflection features and illumination conditions. Finally, by incorporating the two-stage detection framework and the reflection feature regression model, the detection of structurally damaged screws under uneven illumination conditions is realised. The detailed process is illustrated in the following subsections. Table 1 shows the important notations used in this paper.

### 3.1. Characterise Reflection Features

In screw detection tasks, the texture features of screws in EOL products are easily changed by structural damage, which decreases the accuracy of texture features to describe used screws. In this paper, it is proposed to characterise used screws by using reflection features, which are mainly determined by illumination conditions and the object’s reflection abilities as

(1) R=f(P,I)

where f represents the mapping function. With a fixed illumination condition, the difference in reflection features between screws and other components can be regarded as the detection criteria. The reflection ability is highly related to roughness, transparency, and refractive index, while the impact of structural damage on reflection abilities is limited. In addition, the reflection feature of screws is modelled from the point of view of regions, which is also beneficial to reduce the influence of structural damage on feature modelling. In the proposed method, the size of the screw region is determined by the size of the screw in the original products.

### 3.2. Measure Illumination Conditions

**Figure 3:**
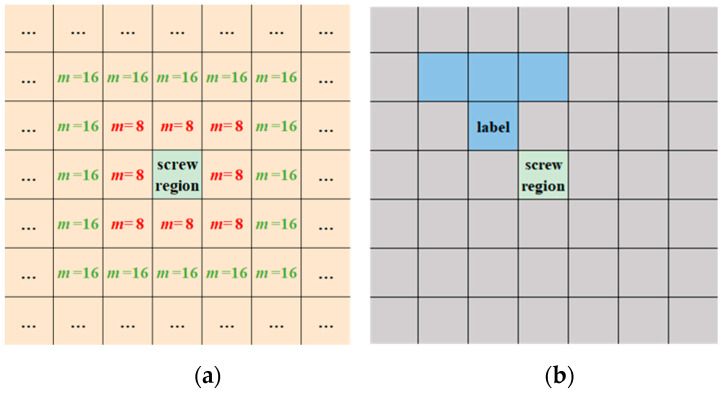
Distribution of the *m*-neighbourhood region of the screw region and the example of an extracted illumination label using Algorithm 1. (**a**) The distribution of the *m*-neighbourhood region of the screw region, where the green rectangle denotes the screw region, and the orange rectangles denote *m*-neighbourhood regions with *m* recorded in the centre (e.g., *m* = 8, *m* = 16). (**b**) An example of an extracted illumination label using Algorithm 1. In (**b**), the green rectangle denotes the screw region, the grey rectangles denote *m*-neighbourhood regions where there are extracted edge features, and the blue rectangles denote *m*-neighbourhood regions where there is no extracted edge feature. The blue rectangle with ‘label’ recorded in the centre denotes the extracted illumination label. The size of each neighbourhood region is the same as the size of the screw region. Due to the adjacent location of the illumination label and the screw region, the illumination condition of the extracted illumination label is the same as the illumination condition of the screw region.

To promote the robustness of reflection features under uneven illumination conditions, the reflection features are expected to be automatically updated based on illumination conditions. The measurement accuracy of illumination conditions determines the representation ability of reflection features. However, it is difficult to set the position of light sensors (e.g., light meters) due to the uncertain and complex image capture environments (e.g., the relative position between cameras and targets, the angular relationship between cameras and targets). Under this situation, the efficiency and accuracy of adopting sensors to measure illumination conditions are unsatisfactory. This paper proposes an illumination label to reflect the illumination condition and describe the relationship between the reflection features of screw regions and illumination conditions from the point of view of images. The introduction of illumination labels is beneficial for accurately and efficiently measuring the illumination condition of screws and is less likely to be affected by complex capture conditions (e.g., shadowing) [30,31,32]. The illumination label extraction algorithm is defined as Algorithm 1, where the position of the screw region ($\left[M_{s},M_{e},N_{s},N_{e}\right]$) is described by its top-left point $\left(M_{s},N_{s}\right)$ and the bottom-right point $\left(M_{e},N_{e}\right)$. Through the illumination label extraction algorithm, the nonfeature region (there are no extracted edge feature points in this region) closest to the screw region is defined as the illumination label.
**Algorithm 1**: illumination label extraction algorithm**Input:**Captured image S;Screw region $S_{m}$;Screw region position $\left[M_{s},M_{e},N_{s},N_{e}\right]$;**Output:**Illumination label $S_{label}$;1:   Use Sobel operators [33] to extract the edge features of captured image S and obtain the edge feature map $S_{f}$;2:   Select the m-neighbourhood region of screw region $S_{m}$ with the same size as the screw region $\left(M_{e}-M_{s},N_{e}-N_{s}\right)$, as shown in Figure 3a;3:   **For** m=8∗loop (loop is initially set to 1) **do**4:     **For** neigh in *m*-neighbourhood regions **do**5:       **If** there is no edge feature in neigh **do**6:         $S_{label}=neigh$;7:         Go to Step 12;8:       **End**9:     **End**10:  loop=loop+1;11:  **End**12:  **Return** $S_{label}$.

The screw region is unknown in the inference process, where we present a self-optimisation strategy to solve this problem, as illustrated in Section 3.4. Figure 3b gives an example of an extracted illumination label by using Algorithm 1.

(2) $$I_{screw}=I_{label}$$

The changes in the illumination condition of the screw region can be represented by the changes in the reflection feature of the illumination label. Therefore, the relationship between the reflection feature of the screw region and the illumination condition is described by the relationship between the reflection feature of the screw region and the reflection feature of the illumination label. In this study, we use the L value in the Lab colour space to represent the illumination conditions and reflection features.

The relationship between the reflection feature of the screw region and the illumination condition is determined by the relationship between $LR_{screw}$ and $LR_{label}$, where $LS_{screw}$ and $LS_{label}$ are first calculated as

(3) $$LS_{screw}=\frac{1}{i*j}\sum_{i=1}^{i=srl}\sum_{j=1}^{j=srw}LS_{screw}(i,j)$$

(4) $$LS_{label}=\frac{1}{i*j}\sum_{i=1}^{i=lrl}\sum_{j=1}^{j=lrw}LS_{label}(i,j)$$

srl and srw denote the size of the screw region.

(5) $$srl=M_{e}-M_{s}$$

(6) $$srw=N_{e}-N_{s}$$

lrl and lrw denote the size of the extracted illumination label, and $LS_{screw}(i,j)$ and $LS_{label}(i,j)$ denote the L value of pixel point (i,j) in the screw region and the illumination label, respectively. Then, $LS_{screw}$ and $LS_{label}$ are divided into reflection components ($LR_{screw}$, $LR_{label}$) and illumination components ($LI_{screw}$, $LI_{label}$) based on retinex theory as

(7) $$LS_{screw}=LR_{screw}•LI_{screw}$$

(8) $$\log(LS_{screw})=\log(LR_{screw})+\log(LI_{screw})$$

(9) $$LS_{label}=LR_{label}•LI_{label}$$

(10) $$\log(LS_{label})=\log(LR_{label})+\log(LI_{label})$$

Finally, by introducing Equation (2), the relationship between $LR_{screw}$ and $LR_{label}$ can be represented by the relationship between $LS_{screw}$ and $LS_{label}$ as

(11) $$\log(LS_{screw})=\log(LR_{screw})+\log(LS_{label})-\log(LR_{label})$$

(12) $$\frac{LS_{screw}}{LS_{label}}=\frac{LR_{screw}}{LR_{label}}$$

Therefore, the relationship between $LR_{screw}$ and $LR_{label}$ is represented by the relationship between $LS_{screw}$ and $LS_{label}$.

### 3.3. Reflection Feature Regression Model

To set the relationship between the reflection feature of the screw region and the reflection feature of the illumination label, a reflection feature regression model is established, where the regression function is determined by $\frac{LR_{screw}}{LR_{label}}$. In deducing the regression function, $LR_{screw}$ and $LR_{label}$ are first expressed based on Equation (1) as

(13) $$LR_{screw}=f(P_{screw},LI_{screw})$$

(14) $$LR_{label}=f(P_{label},LI_{label})$$

Here, the relationship between the reflection ability of the screw region ($P_{screw}$) and the reflection ability of the illumination label ($P_{label}$) can be assumed as

(15) $$P_{screw}=k*P_{label}$$

where k is a constant value. Therefore, $LR_{screw}$ evolves to

(16) $$LR_{screw}=f(P_{screw},LI_{screw})=f(k*P_{label},LI_{label})$$

and f can be described by a polynomial function as

(17) $$f(x)=a_{n}x^{n}+a_{n-1}x^{n-1}+\ldots +a_{0}$$

where $\left[a_{n},a_{n-1},\ldots ,a_{0}\right]$ denotes $LI_{screw}$ and $LI_{label}$, and x denotes $P_{screw}$ and $P_{label}$. By combining Equations (13)–(17), the relationship between $LR_{screw}$ and $LR_{label}$ is confirmed as

(18) $$LR_{label}=a_{n}P_{label}^{n}+a_{n-1}P_{label}^{n-1}+\ldots +a_{0}$$

(19) $$\begin{array}{l} LR_{screw}=a_{n}P_{screw}^{n}+a_{n-1}P_{screw}^{n-1}+\ldots +a_{0}\\ =a_{n}k^{n}P_{label}^{n}+a_{n-1}k^{n-1}P_{label}^{n-1}+\ldots +a_{0} \end{array}$$

where $k^{n},k^{n-1},\ldots ,k$ are the constant value. Considering the constraints of the highest power for $P_{screw}$ ($P_{screw}^{n}$) and $P_{label}$ ($P_{label}^{n}$) and limited training data in industrial tasks, a linear regression model of the reflection feature is constructed as

(20) $$LR_{screw}=w_{r}*LR_{label}+b_{r}$$

aiming to find the optimal weight ($w_{r}$) and bias ($b_{r}$) that can minimise the difference between $a_{n}k^{n}P_{label}^{n}+a_{n-1}k^{n-1}P_{label}^{n-1}+\ldots +a_{0}$ and $a_{n}w_{r}P_{label}^{n}+a_{n-1}w_{r}P_{label}^{n-1}+\ldots +w_{r}a_{0}+b_{r}$ by data learning. It can also be expressed based on Equation (12) as

(21) $$LS_{screw}=w*LS_{label}+b$$

where w and b denote the weight and bias of the linear regression function, respectively. In the following, Equation (21) is adopted to construct the regression model due to the availability of $LS_{screw}$ and $LS_{label}$, where the optimiser used is the Adam optimiser [34] and the loss function is defined as

(22) $$loss=\frac{1}{U}\sum_{i=1}^{U}{\left(LS_{screw_{i}}-LS_{screw_{i}}^{pred}\right)}^{2}$$

where $LS_{screw}^{pred}$ denotes the predicted L value of the screw region and U denotes the data length.

In addition, the above discussion indicates that the reflection features of the screw region and the illumination label can be represented by the L value of the screw region and the illumination label.

### 3.4. Two-Stage Detection Framework

**Figure 4:**
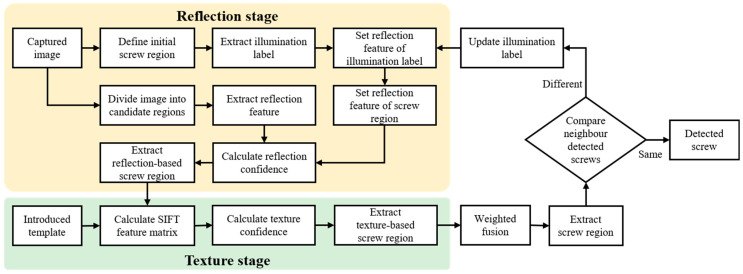
Flowchart of the two-stage detection framework.

With a trained reflection feature regression model, the reflection feature of the screw region is defined and utilised in a proposed two-stage detection framework, which is composed of the reflection stage and the texture stage, as shown in Figure 4.

In the reflection stage, the centre region of the captured image is defined as the initial screw region, and the illumination label is extracted using Algorithm 1. Then, the reflection feature of the illumination label is modelled and used to predict the reflection feature of the screw region with the help of a trained reflection feature regression model. At the same time, the captured image is divided into different candidate regions with the same size as the screw region, while the Euclidean distance between the reflection features of candidate regions and the predicted reflection features of the screw region is calculated [35], named the reflection confidence (CR). Here, each candidate region is assigned a reflection confidence. Finally, rn regions with smaller detection confidences are extracted as reflection-based screw regions.

In the texture stage, the reflection-based screw regions are further analysed based on their texture features, which aims to filter out the false regions. First, the texture features of those reflection-based screw regions are detected by using the SIFT descriptor [36], where a SIFT feature matrix of each reflection-based screw region (B) is constructed. Then, by calculating the SIFT feature matrix of an introduced screw template (T), the distance (D) in SIFT feature matrices between the reflection-based screw region and the screw template is obtained as

(23) $$D(x,y)=\sqrt{\sum_{i=1}^{i=sn}{\left(T(x,i)-B(y,i)\right)}^{2}},x\in [1,p],y\in [1,q]$$

where T is a {p,sn} feature matrix, B is a {q,sn} feature matrix, and D is a {p,q} feature matrix. D(x,y), T(x,y), and B(x,y) denote the value of point (x,y) for D, T, and B, respectively. p and q denote the number of extracted feature points from the introduced screw template and the reflection-based screw region, respectively, while each extracted feature point is described by a {sn} vector. Based on the distance matrix, a texture confidence matrix (CT) for each reflection-based screw region is expressed as

(24) $$CD(x,y)=D(x,y)/\sum_{i=1}^{i=p}D(i,y),x\in [1,p],y\in [1,q]$$

(25) CT(x)=min(CD[x,:]),x∈[1,p]

where CD records the ratio between different texture feature distances, CT is a {p} vector, and CT(x) denotes the value of point (x) for matrix CT. Finally, the texture confidence (CT) for each reflection-based screw region is computed as

(26) $$CT=\sum_{i=1}^{i=p}CT(i)$$

and tn regions with smaller texture confidences are extracted as texture-based screw regions.

Through the reflection stage and the texture stage, tn texture-based screw regions are extracted, and each extracted region is assigned a reflection confidence and a texture confidence. Then, the total reflection confidence (totalCR) and the total texture confidence (totalCT) are calculated by adding up these tn reflection confidences and texture confidences, respectively. Finally, each texture-based screw region is assigned a fused detection confidence (C) as

(27) $$C=\frac{CR}{2*totalCR}+\frac{CT}{2*totalCT}$$

The screw region with the lowest fused detection confidence is extracted and named the detected screw.

In addition, the initial screw region is randomly defined, and thus, the corresponding extracted illumination label cannot be used to determine the reflection features of the screw region in the inference process. A self-optimisation strategy is proposed to continuously update the screw region and illumination label by forming the reflection stage and the texture stage into a loop, where the difference in detection results between the neighbouring iterations is employed as a judgement.

(28) $$\left\{ \begin{array}{l} \left|M_{s}^{t}-M_{s}^{t+1}\right|/M_{s}^{t+1}\leq td\\ \left|M_{e}^{t}-M_{e}^{t+1}\right|/M_{e}^{t+1}\leq td\\ \left|N_{s}^{t}-N_{s}^{t+1}\right|/N_{s}^{t+1}\leq td\\ \left|N_{e}^{t}-N_{e}^{t+1}\right|/N_{e}^{t+1}\leq td \end{array}\right.:\mathrm{no}\;\mathrm{difference}$$

where td is a given threshold. If there is no difference, the detected screw of the current iteration will be accepted as the final detected screw. Otherwise, this detected screw is regarded as a falsely detected screw and would be utilised to update the illumination label by using Algorithm 1 for the new iteration.

By integrating the reflection feature regression model and the two-stage detection framework, a screw detection method is achieved for detecting structurally damaged screws in EOL products under nonuniform illumination conditions.

## 4. Experiments

### 4.1. Experimental Setup

**Figure 5:**
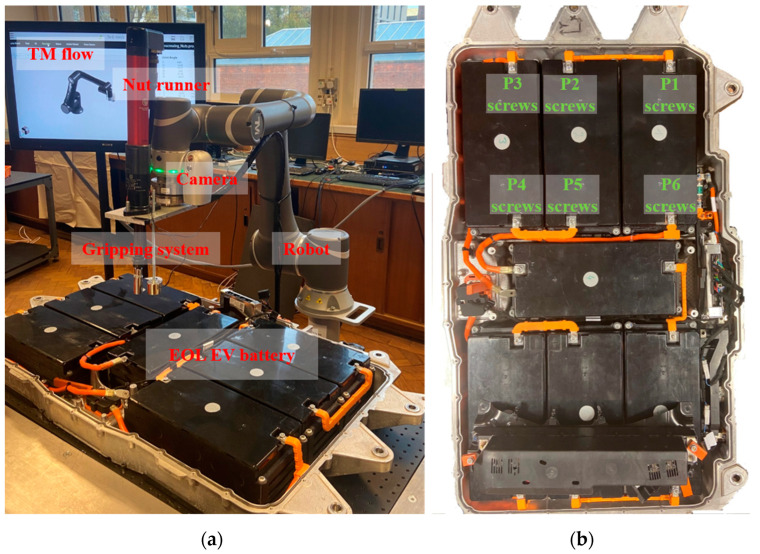
Experimental platform. (**a**) Utilised equipment; (**b**) EOL plug-in hybrid EV battery and the position of adopted screws in the experiments.

The proposed screw detection method was implemented in removing used screws from an EOL plug-in hybrid EV battery in a robotic disassembly platform, as shown in Figure 5. The utilised equipment contains an industrial robot, a 2D industrial camera, an electrical nut runner, and an electromagnetic gripping system. The control of the above equipment was achieved by programming on TM flow v1.82 software [37], while the detection method was realised through Python v3.8 and MATLAB v9.1 programming on an equipped workstation.

### 4.2. Experimental Procedure

**Figure 6:**
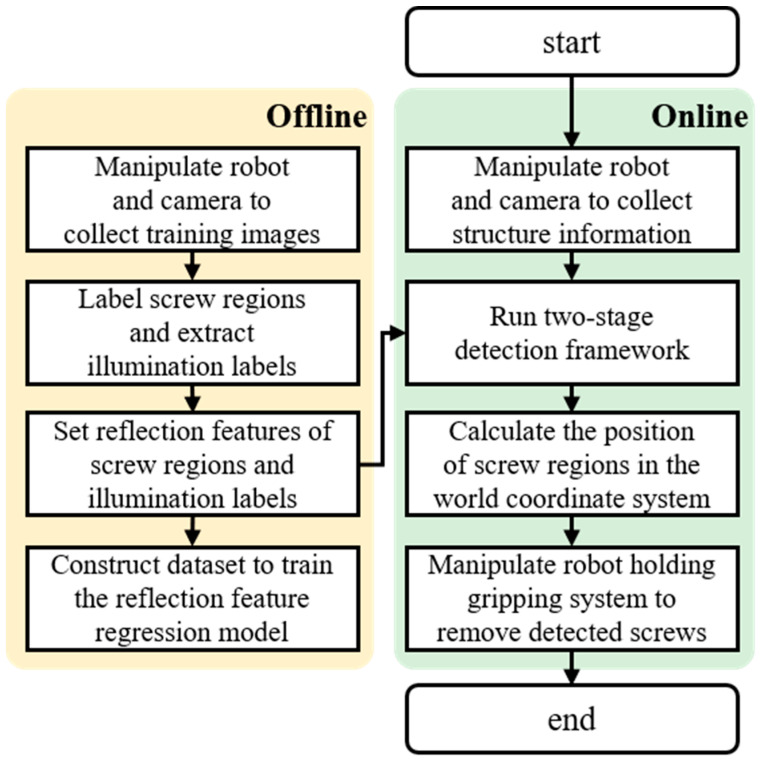
Flowchart of the experimental procedure.

Considering the training process of the designed reflection feature regression model, an experimental procedure containing an offline process and an online process was developed, as shown in Figure 6. In the offline process, a robot holding a camera was used to collect training images. The screw regions in the collected images were labelled by human operators and then utilised to extract illumination labels by running Algorithm 1. Finally, the reflection features of screw regions and illumination labels were calculated to construct the dataset for training the reflection feature regression model. In the online process, a robot holding a camera was also employed to collect the structural information of the screws used. Next, by inputting the trained reflection feature regression model and captured image to the two-stage detection framework, the locations of screw regions in the image coordinate system were obtained. Finally, by transforming the position of screw regions in the image coordinate system to the position in the world coordinate system, the robot holding an electromagnetic gripping system was able to remove detected screws from an EOL plug-in hybrid EV battery.

In the experiments, the removal of hexagonal-headed screws (M6 nuts) in 6 different positions was used as an example to discuss the detection performance. The distribution of the 6 positions is shown in Figure 5b, while the screws located in these positions are named P1 screws, P2 screws, P3 screws, P4 screws, P5 screws, and P6 screws. In addition, to quantitatively validate the detection performance, 9 training datasets and 40 test datasets were constructed for P1 screws, P2 screws, P3 screws, P4 screws, P5 screws, and P6 screws. The numbers of images in one training dataset and one test dataset are 200 and 50, respectively. The data collection was conducted under various illumination conditions, and the condition of screws (e.g., the degree of structural damage) was updated constantly during the collection process. Table 2 records the experimental parameters of the detection method in the screw removal case.

### 4.3. Evaluation System

The detection performance was first evaluated based on the mean average precision (mAP) [38] at different values of the interaction over union (IOU) between the detected screw region $S_{d}$ and the actual screw region $S_{r}$. Here, IOU is calculated as

(29) $$IOU=\left(S_{d}\cap S_{r}\right)/\left(S_{d}\cup S_{r}\right)$$

As the most commonly used evaluation indicator in the object detection field, mAP can reflect the performance of the proposed screw detection method considering recall and precision.

Then, the operation range of the designed socket in the gripping system was taken into account, and the proposed detection method based on the disassembly accuracy (accuracy) was evaluated as

(30) accuracy=T/(T+F)

where the centre of the operation range is the same as the centre of the detected screw region. T is the number of samples whose actual screw region is completely covered by the operation range, and F is the number of samples whose actual screw region is not completely covered by the operation range. By introducing the operation range of sockets, the success rate of unfastening screws based on the screw detection results can be evaluated using accuracy.

Apart from analysing the performance of the proposed detection method, the R-squared value was also used to validate the goodness-of-fit of the developed reflection feature regression model [39]. The range of the R-squared value is from 0 to 1, and a higher value indicates a better fitting performance. The performance of the reflection feature regression model is closely related to the stability of the proposed screw detection method. When the reflection feature regression model achieves satisfactory fitting performance represented by a high R-squared value, the proposed screw detection method is more likely to accurately and stably detect screws under uneven illumination conditions.

## 5. Results and Discussion

### 5.1. Performance of the Reflection Feature Regression Model

**Figure 7:**
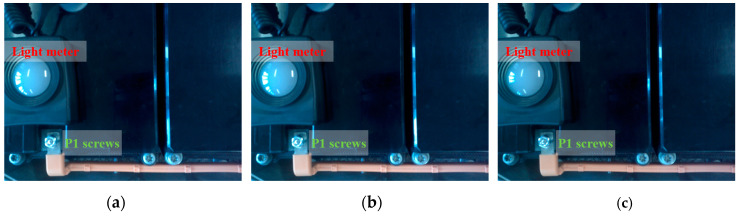
Example captured images for P1 screws. (**a**–**c**) The images captured in three different positions at the same time.

To evaluate the fitting performance of the reflection feature regression model, the capability of using illumination labels to reflect illumination conditions for P1 screws was first analysed. Figure 7 shows the images captured in three different positions, and Table 3 tabulates the reflection features of screws and the illumination conditions of screws measured by light meters and illumination labels. The capture positions for the three images are different from each other, and the reflection features of screws ($LS_{screw}$) in the three images are 58.4615, 59.3731, and 56.8118. The difference in reflection features indicates that the screw was captured under different illumination conditions. However, the light meters cannot measure the difference and obtain the same value of illumination conditions (770 lx). Using the illumination label, the tiny differences in illumination conditions were successfully detected, where the illumination conditions of screws ($LS_{label}$) are 14.7876, 14.9791, and 12.1517. With the satisfactory performance of measuring illumination conditions, the relationship between the reflection features of screw regions and the illumination conditions can be accurately described by the relationship between the reflection features of screw regions and the reflection features of illumination labels.

**Figure 8:**
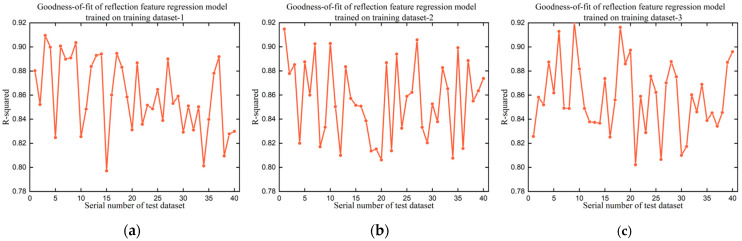

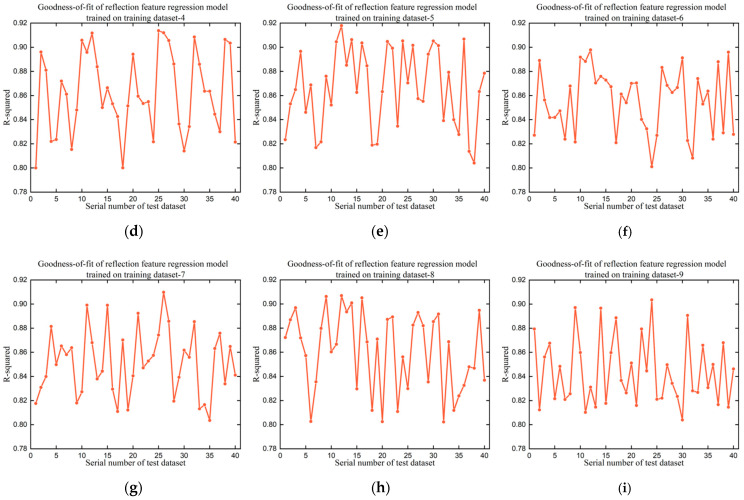
Goodness-of-fit of 9 trained reflection feature regression models on 40 test datasets for P1 screws. (**a**–**i**) The R-squared value of the reflection feature regression model trained on training dataset-1, training dataset-2, training dataset-3, training dataset-4, training dataset-5, training dataset-6, training dataset-7, training dataset-8, and training dataset-9, respectively.

In validating the reflection feature regression model, the reflection feature regression model was trained on 9 training datasets, and the 9 trained models were then tested on 40 test datasets for P1 screws. Figure 8 shows the detailed R-squared value and Table 4 records the maximum value, minimum value, and average value. In the experiments, the maximum R-squared value, the minimum R-squared value, and the average R-squared value are approximately 0.91, 0.80, and 0.86, respectively, which reflects the excellent fitting ability of the proposed reflection feature regression model. The small differences in the maximum R-squared value, the minimum R-squared value, and the average R-squared value among the nine subgraphs confirm the satisfactory robustness of the proposed reflection feature regression model.

Overall, the proposed reflection feature regression model performed well in establishing the relationship between the reflection features of screw regions and the reflection features of illumination labels under uneven illumination conditions. This attractive fitting performance is mainly contributed by the suitable linear regression function, which was deduced in designing the reflection feature regression model. In the following experiments, the reflection feature regression model trained on training dataset-1 was used to determine the reflection features of screw regions in running the two-stage detection framework for P1 screws.

### 5.2. Performance of the Two-Stage Detection Framework

**Figure 9:**
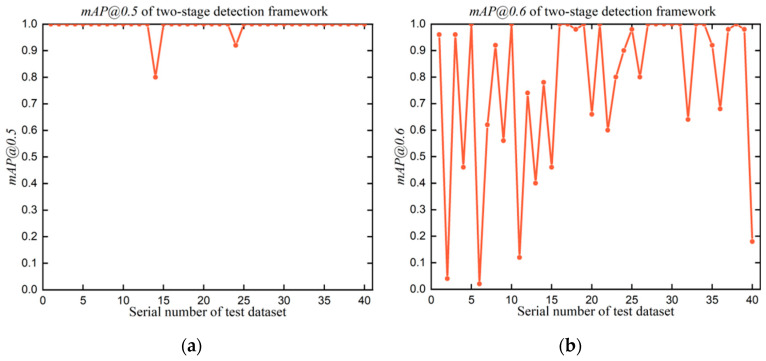

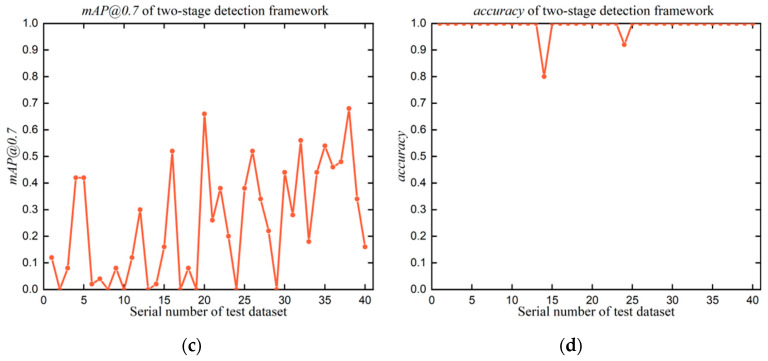
Detection performance of the proposed two-stage detection framework for P1 screws. (**a**–**d**) The detection performance evaluated by mAP@0.5, mAP@0.6, mAP@0.7, and accuracy, respectively.

The determined reflection features were adopted to extract screw regions together with texture features in the designed two-stage detection framework. The output of the two-stage detection framework is the final result of the proposed screw detection method. Figure 9 and Table 5 validate the detection performance of the proposed two-stage detection framework on 40 test datasets for P1 screws in terms of mAP@IOU (IOU=0.5, 0.6, 0.7) and accuracy. The maximum mAP@0.5, minimum mAP@0.5, and average mAP@0.5 are 1.00, 0.80, and 0.99, respectively, which demonstrate the reliable detection performance of the two-stage detection framework. The maximum mAP@0.6, minimum mAP@0.6, and average mAP@0.6 are 1.00, 0.02, and 0.78, respectively, while the maximum mAP@0.7, minimum mAP@0.7, and average mAP@0.7 are 0.68, 0.00, and 0.25, respectively. The downward trend from mAP@0.5 to mAP@0.7 is caused by the stricter settings in IOU. In addition, Figure 9d and Table 5 show the detection performance based on accuracy. Here, the maximum accuracy, minimum accuracy, and average accuracy are 1.00, 0.80, and 0.99, respectively. The satisfactory accuracy proves that the two-stage detection framework enabled the screw removal tasks to be operated accurately under nonuniform illumination conditions. The remarkable detection capability is granted by the integration of reflection features and texture features, which has stronger representation ability for structurally damaged screws. On the other hand, no significant difference was found in evaluating the detection performance between using mAP@0.5 and accuracy. Under this situation, it was concluded that mAP@0.5 is more suitable for evaluating the detection performance compared with mAP@0.6 and mAP@0.7 in the experimental case. In the following experiments, the detection performance was mainly discussed based on mAP@0.5 and accuracy.

**Figure 10:**
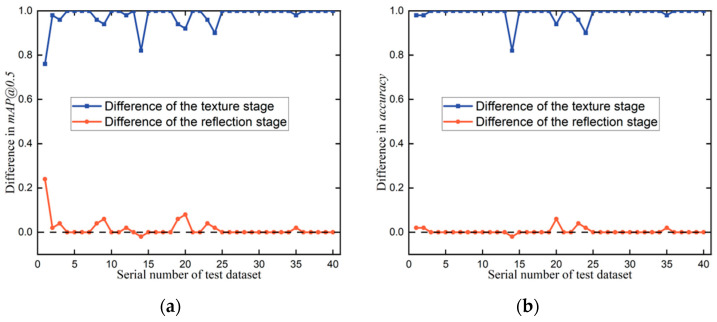
Contributions of the reflection stage and the texture stage to the two-stage detection framework for P1 screws. The blue lines record the differences between using the two-stage detection framework and using the texture stage, while the red lines record the differences between using the two-stage detection framework and using the reflection stage. (**a**) Difference in mAP@0.5; (**b**) difference in accuracy.

To study the contribution of reflection features and texture features to the two-stage detection framework, the reflection stage and the texture stage were used to detect P1 screws on the 40 test datasets. Figure 10 and Table 6 compare the difference in mAP@0.5 and accuracy between using the two-stage detection framework and using the reflection stage and between using the two-stage detection framework and using the texture stage. A higher difference denotes that the stage makes fewer contributions to the detection framework. As shown in Table 6, the maximum difference, minimum difference, and average difference in mAP@0.5 between using the two-stage detection framework and the texture stage are 1.00, 0.76, and 0.98, while the maximum difference, minimum difference, and average difference in mAP@0.5 between using the two-stage detection framework and the reflection stage are 0.24, −0.02, and 0.02, respectively.

These results demonstrate that the satisfactory detection performance of the two-stage detection framework is mainly achieved by using reflection features. Specifically, there are some points with a mAP@0.5 value of 1.00 in the difference between using the two-stage detection framework and using the texture stage and some points with a mAP@0.5 value of 0.00 in the difference between using the two-stage detection framework and using the reflection stage. In the testing experiments corresponding to these points, accurate detection is completely accomplished by the reflection stage.

Furthermore, some points were found with negative mAP@0.5 values in the difference between using the two-stage detection framework and using the reflection stage. Here, the utilisation of the texture stage limited the detection capability of the two-stage detection framework. The experimental conclusions drawn from Figure 10b are consistent with those concluded from Figure 10a. In conclusion, the reflection stage is a major contributor, and the texture stage is an optimiser in the two-stage detection framework. This is caused by the properties of structurally damaged screws in EOL products. As discussed before, the texture features of screws used in EOL products are not reliable because they are easily damaged, while the representation capability of reflection features for used screws is more accurate and robust. The significant contribution of the reflection stage to the two-stage detection framework also indicates the satisfactory measuring ability of illumination labels and the remarkable learning capability of the reflection feature regression model.

### 5.3. Generalisation

Considering the impact of background information on screw detection, it was decided to retest the proposed screw detection method comprising the reflection feature regression model and the two-stage detection framework with P2 screws, P3 screws, P4 screws, P5 screws, and P6 screws, as recorded in Table 7. In the experiments, the testing environments (e.g., illumination conditions, capture positions), object conditions (e.g., the degree of structural damage, the level of tightness), and surrounding objects of each testing experiment are different from each other.

The average mAP@0.5 (0.91) and accuracy (0.91) confirm the satisfactory detection performance under various illumination conditions. The small fluctuations in mAP@0.5 and accuracy among the six experiments show the robustness of the proposed detection method. In addition, the mAP@0.5 and accuracy for P4 screws, P5 screws, and P6 screws are lower than those for P1 screws, P2 screws, and P3 screws. This is because the testing environment, object conditions, and surrounding objects are more complex, which creates more false screw regions. However, their performance is also acceptable. In conclusion, the proposed detection method realised accurate and stable detection for structurally damaged screws in EOL products under uneven illumination conditions, where the reflection feature regression model empowered the reflection feature to be adjusted automatically and the two-stage detection framework integrated the reflection information and texture information to comprehensively and accurately characterise the screws used. Table 8 summarises the performance of the reflection feature regression model and the two-stage detection framework.

### 5.4. Comparison

The proposed screw detection method was also compared with existing methods, as shown in Table 9. As mentioned before, the existing screw detection methods are generally divided into experience-based methods and data-driven methods. In experience-based methods, the detection criteria are designed based on the specific properties of different EOL objects. It is not reasonable to adopt existing experience-based methods in this case and compare their performance directly. Here, the detection performance of various feature descriptors utilised in [15,16,17] was tested, and the optimal mAP@0.5 and accuracy were recorded. In addition, the optimal detection performance of the aforementioned data-driven methods [18,19,20,21] was also recorded, where YOLO [20] achieved higher mAP@0.5 and accuracy.

As shown in Table 9, the proposed screw detection method performed much better than the existing experience-based methods, which mainly benefits from the adoption of reflection features and the reflection feature regression model. The mAP@0.5 and accuracy have been significantly improved by 0.81 and 0.74. The experience-based methods are still used in various disassembly tasks due to the lower requirement for labelled data. The proposed screw detection method also performed better than some data-driven methods, such as Soar, ResNet, and faster R-CNN. The increases in mAP@0.5 are 0.15, 0.11, and 0.04, respectively, and the increases in accuracy are 0.13, 0.11, and 0.04, respectively. Although YOLO performed slightly better than the proposed method (the differences in mAP@0.5 and accuracy are 0.03 and 0.03), the demanding requirement of YOLO for training data poses great challenges to screw removal tasks, as shown in Table 10. The mAP@0.5 of the YOLO model trained on 200 images is 0.23, which is significantly lower than the mAP@0.5 of the proposed method trained on 200 images. The proposed screw detection method is not greedy for training data due to the development of the lightweight reflection feature regression model and the integration of human experience and training data in the designed two-stage detection framework. The demanding requirement of complex deep learning models (e.g., YOLO) for training data can be partially addressed by transfer learning. However, the detection performance is closely related to the diversity of training samples. The collected training samples are expected to provide a comprehensive characterisation of the structural information for unseen used screws, which is impossible due to the uncertainties of EOL products. The proposed screw detection method adopts reflection features to deal with the uncertainties in the structural information and obtained robust detection performance. Consequently, the proposed screw detection method is reliable to help accomplish complex screw removal tasks under uneven illumination conditions.

## 6. Conclusions and Future Work

Research on screw detection is essential for automatic removal. This paper realises the aim of accurately and stably detecting structurally damaged screws in EOL products under uneven illumination conditions by presenting a reflection feature regression model and a two-stage detection framework. The utilisation of reflection features addresses the problem of characterising damaged screw structures caused by the uncertainty and complexity of EOL products, and the reflection features are automatically determined by data learning according to the illumination conditions. The texture feature helps the proposed screw detection method filter out falsely detected screws. In addition, an innovative illumination condition measurement method is proposed in employing the reflection feature by defining the illumination label. The detection method is optimised by a developed self-optimisation strategy. Finally, a vision-guided robotic disassembly platform designed to disassemble EV batteries is utilised to evaluate the detection performance. This study realises stable and accurate screw detection on a battery disassembly task and contributes to the research and application of object detection in other disassembly cases.

The developed method has two issues requiring research in the future. First, this study takes the influence of structural damage on screw detection into account. However, the problem of detecting rusty screws needs to be researched further. Future work plans to optimise the proposed method by designing a stronger data-driven network to describe the reflection characteristics of rusty screws. Second, the proposed illumination condition measurement method lacks a quantitative assessment. Future work plans to adopt sensors to describe the trend of illumination conditions to reflect the performance of the proposed measurement method.

## Figures and Tables

**Table 1:** The important notations used in this paper.

Notation	Description
R	Reflection features
P	Reflection abilities
I	Illumination conditions
$I_{screw}$	The illumination condition of the screw region
$I_{label}$	The illumination condition of the illumination label
$LR_{screw}$	The L value of the reflection component for the screw region
$LR_{label}$	The L value of the reflection component for the illumination label
$LS_{screw}$	The L value of the screw region
$LS_{label}$	The L value of the illumination label
$LI_{screw}$	The L value of the illumination component for the screw region
$LI_{label}$	The L value of the illumination component for the illumination label
$P_{screw}$	The reflection ability of the screw region
$P_{label}$	The reflection ability of the illumination label

The L values of the reflection components for the screw region and the illumination label are used to represent the reflection features of the screw region and the illumination label, while the illumination conditions of the screw region and the illumination label are recorded by the L values of the illumination components for the screw region and the illumination label. The L values are extracted from the Lab colour space in this study.

**Table 2:** Experimental parameters of the detection method in the screw removal case.

The Length of the Screw Region (srl)	The Width of the Screw Region (srw)	The Number of Extracted Reflection-Based Screw Regions (rn)	The Size of the Extracted Texture Feature Point (sn)	The Number of Extracted Reflection-Based Screw Regions (tn)	The Difference Threshold for Comparing Neighbouring Detected Screws (td)
100	100	20	128	10	0.05

**Table 3:** Performance of measuring illumination conditions by light meters and illumination labels for P1 screws.

	Capture Position (X, Y, Z, Rx, Ry, Rz)	$LS_{screw}$	$LS_{label}$	Illumination Conditions Measured by Light Meters
Figure 7a	(140 mm, −534 mm, 424 mm, 179°, −3°, −8°)	58.4615	14.7876	770 lx
Figure 7b	(160 mm, −534 mm, 424 mm, 179°, −3°, −8°)	59.3731	14.9791	770 lx
Figure 7c	(140 mm, −515 mm, 424 mm, 179°, −3°, −8°)	56.8118	12.1517	770 lx

The adopted light meter is CA 1110 developed by Chauvin Arnoux. Measurement range: 0.1 lx to 200,000 lx; ±3% of reading on incandescent sources; ±6% of the reading on LEDs; ±9% of the reading on fluorescent sources.

**Table 4:** The maximum, minimum, and average R-squared values of 9 trained reflection feature regression models on 40 test datasets for P1 screws.

	Maximum R-Squared	Minimum R-Squared	Average R-Squared
Training dataset-1	0.909	0.797	0.860
Training dataset-2	0.915	0.806	0.856
Training dataset-3	0.920	0.802	0.858
Training dataset-4	0.914	0.800	0.862
Training dataset-5	0.918	0.804	0.867
Training dataset-6	0.898	0.801	0.855
Training dataset-7	0.910	0.804	0.851
Training dataset-8	0.907	0.802	0.861
Training dataset-9	0.904	0.804	0.844

**Table 5:** The maximum, minimum, and average mAP@0.5, mAP@0.6, mAP@0.7, and accuracy of the two-stage detection framework for P1 screws.

	Maximum Value	Minimum Value	Average Value
mAP@0.5	1.00	0.80	0.99
mAP@0.6	1.00	0.02	0.78
mAP@0.7	0.68	0.00	0.25
accuracy	1.00	0.80	0.99

**Table 6:** The maximum, minimum, and average differences in mAP@0.5 and accuracy between using the two-stage detection framework and using the texture stage and between using the two-stage detection framework and using the reflection stage.

	Difference between Using the Two-Stage Detection Framework and Using the Texture Stage	Difference between Using the Two-Stage Detection Framework and Using the Reflection Stage
Maximum mAP@0.5	1.00	0.24
Minimum mAP@0.5	0.76	−0.02
Average mAP@0.5	0.98	0.02
Maximum accuracy	1.00	0.06
Minimum accuracy	0.82	−0.02
Average accuracy	0.99	0.01

**Table 7:** mAP@0.5 and accuracy of the proposed detection method on different screws.

	mAP@0.5	accuracy
P1 screws	0.99	0.99
P2 screws	0.97	0.97
P3 screws	0.97	0.97
P4 screws	0.82	0.82
P5 screws	0.84	0.84
P6 screws	0.86	0.86
average	0.91	0.91

**Table 8:** The performance of the reflection feature regression model and the two-stage detection framework.

	Average R-Squared	Average mAP@0.5	Average accuracy
Reflection feature regression model	0.857	\	\
Two-stage detection framework	\	0.91	0.91

**Table 9:** Comparison of the detection performance with existing methods.

	mAP@0.5	accuracy
Experience-based methods [15,16,17]	0.10	0.17
Soar [18]	0.76	0.78
ResNet [19]	0.80	0.80
YOLO [20]	0.94	0.94
Faster R-CNN [21]	0.87	0.87
Proposed	0.91	0.91

**Table 10:** Number of utilised training data in the proposed screw detection method and existing data-driven methods.

	YOLO [20]	Proposed
Number of used training data	6720	200

## Data Availability

The data in this study are available from the corresponding author upon request.
